# Framework for Managing the COVID-19 Infodemic: Methods and Results of an Online, Crowdsourced WHO Technical Consultation

**DOI:** 10.2196/19659

**Published:** 2020-06-26

**Authors:** Viroj Tangcharoensathien, Neville Calleja, Tim Nguyen, Tina Purnat, Marcelo D’Agostino, Sebastian Garcia-Saiso, Mark Landry, Arash Rashidian, Clayton Hamilton, Abdelhalim AbdAllah, Ioana Ghiga, Alexandra Hill, Daniel Hougendobler, Judith van Andel, Mark Nunn, Ian Brooks, Pier Luigi Sacco, Manlio De Domenico, Philip Mai, Anatoliy Gruzd, Alexandre Alaphilippe, Sylvie Briand

**Affiliations:** 1 International Health Policy Program Ministry of Public Health Nonthaburi Thailand; 2 Directorate for Health Information and Research Ministry for Health Valetta Malta; 3 High Impact Events Preparedness Global Infectious Hazards Preparedness Emergency Preparedness Geneva Switzerland; 4 Department of Digital Health and Innovation Science Division World Health Organization Geneva Switzerland; 5 Information Systems for Health Evidence and Intelligence for Action in Health Pan American Health Organization and World Health Organization Regional Office for the Americas Washington DC, DC United States; 6 Evidence and Intelligence for Action in Health Pan American Health Organization and World Health Organization Regional Office for the Americas Washington, DC United States; 7 Department of Health Systems Development Regional Office for South-East Asia World Health Organization New Delhi India; 8 Department of Science, Information and Dissemination Eastern Mediterranean Regional Office World Health Organization Cairo Egypt; 9 Division of Health Systems and Public Health Regional Office for Europe World Health Organization Copenhagen Denmark; 10 Communications Department Regional Office for Africa World Health Organization Brazzaville Congo; 11 Global Infectious Hazards Preparedness Emergency Preparedness World Health Organization Geneva Switzerland; 12 Center for Health Informatics School of Information Sciences University of Illinois Champaign, IL United States; 13 Fondazione Bruno Kessler Trento Italy; 14 Venice Office Organisation for Economic Co-operation and Development Venice Italy; 15 The University Institute for Modern Languages Milan Italy; 16 Berkman-Klein Center for Internet and Society Harvard University Cambridge, MA United States; 17 Complex Multilayer Networks (CoMuNe) Research Center for Information and Communication Technology Bruno Kessler Foundation Trento Italy; 18 Ted Rogers School of Management Ryerson University Toronto, ON Canada; 19 EU DisinfoLab Brussels Belgium

**Keywords:** COVID-19, infodemic, knowledge translation, message amplification, misinformation, information-seeking behavior, access to information, information literacy, communications media, internet, risk communication, evidence synthesis

## Abstract

**Background:**

An infodemic is an overabundance of information—some accurate and some not—that occurs during an epidemic. In a similar manner to an epidemic, it spreads between humans via digital and physical information systems. It makes it hard for people to find trustworthy sources and reliable guidance when they need it.

**Objective:**

A World Health Organization (WHO) technical consultation on responding to the infodemic related to the coronavirus disease (COVID-19) pandemic was held, entirely online, to crowdsource suggested actions for a framework for infodemic management.

**Methods:**

A group of policy makers, public health professionals, researchers, students, and other concerned stakeholders was joined by representatives of the media, social media platforms, various private sector organizations, and civil society to suggest and discuss actions for all parts of society, and multiple related professional and scientific disciplines, methods, and technologies. A total of 594 ideas for actions were crowdsourced online during the discussions and consolidated into suggestions for an infodemic management framework.

**Results:**

The analysis team distilled the suggestions into a set of 50 proposed actions for a framework for managing infodemics in health emergencies. The consultation revealed six policy implications to consider. First, interventions and messages must be based on science and evidence, and must reach citizens and enable them to make informed decisions on how to protect themselves and their communities in a health emergency. Second, knowledge should be translated into actionable behavior-change messages, presented in ways that are understood by and accessible to all individuals in all parts of all societies. Third, governments should reach out to key communities to ensure their concerns and information needs are understood, tailoring advice and messages to address the audiences they represent. Fourth, to strengthen the analysis and amplification of information impact, strategic partnerships should be formed across all sectors, including but not limited to the social media and technology sectors, academia, and civil society. Fifth, health authorities should ensure that these actions are informed by reliable information that helps them understand the circulating narratives and changes in the flow of information, questions, and misinformation in communities. Sixth, following experiences to date in responding to the COVID-19 infodemic and the lessons from other disease outbreaks, infodemic management approaches should be further developed to support preparedness and response, and to inform risk mitigation, and be enhanced through data science and sociobehavioral and other research.

**Conclusions:**

The first version of this framework proposes five action areas in which WHO Member States and actors within society can apply, according to their mandate, an infodemic management approach adapted to national contexts and practices. Responses to the COVID-19 pandemic and the related infodemic require swift, regular, systematic, and coordinated action from multiple sectors of society and government. It remains crucial that we promote trusted information and fight misinformation, thereby helping save lives.

## Introduction

A pneumonia of unknown cause detected in Wuhan, China was first reported to the World Health Organization (WHO) Country Office in China on December 31, 2019. The disease, caused by a novel coronavirus, was subsequently named the coronavirus disease (COVID-19) and declared a Public Health Emergency of International Concern on January 30, 2020. On March 11, 2020, the WHO characterized the outbreak as a pandemic. As of June 2, 2020, more than 6.1 million cases of COVID-19 had been reported in over 200 countries and territories, resulting in more than 373,500 deaths, as reported to the WHO [1].

On February 15, 2020, the WHO Director-General Tedros Adhanom Ghebreyesus raised the concern that the epidemic was accompanied by an infodemic [2]. An infodemic is an overabundance of information—some accurate and some not—that occurs during an epidemic [3]. In a similar manner to an epidemic, it spreads between humans via digital and physical information systems. It makes it hard for people to find trustworthy sources and reliable guidance when they need it [4]. People need accurate information during epidemics to modify their behavior and protect themselves, their families, and their communities against the infection. An infodemic cannot be eliminated, but it can be managed. The management of an infodemic becomes more challenging with social media and the rapid spread of information. Similar to epidemics, the information spreads further and faster, propagated by the interconnected ways in which information is disseminated and consumed through the web, digital and social media, chat apps, TV, radio, and other communication channels.

In the context of the COVID-19 pandemic, the infodemic is exacerbated by the global scale of the emergency. Importantly, even though some misinformation may just be confusing, many false and misleading claims such as those about fake or questionable cures, or incorrect recommendations about prevention or public behavior can be harmful to life and can exacerbate the outbreak. The infodemic can be even more challenging to manage when health information messages and facts are incorporated into political narratives and online commentary that is not grounded in verified facts and evidence.

At the same time, however, with the vast amount of information related to the spread of the virus, online and offline interactions, and public opinions expressed on social media can also be valuable sources of knowledge when analyzing the dynamics of the pandemic. For instance, they can help us gauge public sentiment toward different public health measures, analyze adherence to prevention approaches, develop effective public health campaigns, track and map symptoms, predict outbreaks, and detect and combat misinformation. Big data sources and unstructured text data sets can be used alongside official and established ones for real time analytics and modeling as part of infodemic and pandemic management—although such use cases for data also raise questions related to privacy, security, and the ethics of using public and private data sets.

To counter and understand the rapidly changing landscape of the COVID-19 infodemic and develop effective strategies to mitigate its negative side effects such as the spread of misinformation, on April 7 and 8, 2020, the WHO Information Network for Epidemics (EPI-WIN) organized a 2-day global online consultation on managing the infodemic. The meeting materials, recordings of sessions, and summary illustrations of discussion are available on the WHO website [5]. This paper summarizes the proceedings and outcomes of the consultation and recommendations for further action by the WHO, its Member States, and other stakeholders.

## Methods

The aim of this consultation was to crowdsource ideas to form a novel COVID-19 infodemic response framework. A 2-day online consultation with four plenary sessions and a brainstorming session was conducted entirely online using three information and communication platforms: Zoom, Slido, and Twitter. The plenary sessions used the Zoom platform for 19 talks by 21 presenters and 3 discussants. The speakers were identified based on discussion among key staff in the WHO COVID-19 infodemic management response pillar and a search of academic and grey literature for authors in key topics of interest.

Invitations to participate in the consultation were sent out through existing global and regional networks with the aim of bringing together a community of key partners already working to address the infodemic from different perspectives, including but not limited to risk communication, health information systems, research and science, policy analysis, evidence synthesis, digital health, community response, and humanitarian response. All participants were also encouraged to post their thoughts and engage with the wider audience using Twitter and the hashtag #infodemicCOVID19.

The first panel of the meeting consisted of speakers who outlined key perspectives on the infodemic from the points of view of the WHO, national health policy makers, institutes of public health, the news and media, social media platforms, the private sector, publishers of scientific journals, sociobehavioral science, and civil society. The speakers on subsequent panels presented relevant methods, tools, and evidence from past and current experiences dealing with the COVID-19 infodemic. The talks covered a wide range of topics, including risk communication and community engagement in outbreaks; fact-checking practices; identification and response strategies for misinformation; sociobehavioral science research methods; and the use of social listening, artificial intelligence (AI), and computational methods to produce insights for infodemic response decision making. The recommendations from the talks were factored into the subsequent analysis of the actions proposed by the wider group of online participants.

Only panelists and moderators were able to use audio during the Zoom webinar. In parallel, questions were submitted by participants through Slido, a question and answer as well as polling platform. Participants were able to upvote relevant questions and discuss the questions with each other by replying to the questions and comments posted on Slido. Moderators selected the three or four most upvoted questions at the end of each session to pose to panelists. Plenary sessions ran each day from 2 PM to 5 PM Central European Time (CET) to facilitate participation from a wide range of different time zones.

Slido was also used to crowdsource ideas from participants and panelists concerning the elements of a future infodemic management framework. Four breakout topics were established for which ideas could be submitted using Slido from 3 PM CET on April 7, 2020, to 12 PM CET on April 9, 2020. For the final meeting session on April 8, organizers prepared a preliminary narrative analysis of submitted ideas and organized them into subcategories of four thematic areas. For each subcategory, the most frequently named and most innovative ideas were collected for narrative categorization and summary. The selection of ideas was based on the frequency with which an idea was mentioned, the number of likes and replies it generated via Slido, and a qualitative analysis by the WHO analysis team. During the review of the submissions, similar proposals were merged into combined suggestions, and multi-thematic submissions were included in the relevant thematic areas of analysis. Two invited policy makers from the Ministry of Health of Thailand and the Ministry for Health of Malta received the preliminary summary of the narrative 90 minutes before the final session so that they could respond to it with their perspectives and comments. After the submission period for ideas closed on April 9, a final narrative analysis was conducted for all ideas submitted on the platform, including through meeting comments. That summary forms the results of this paper.

After the consultation concluded, the WHO EPI-WIN team conducted an after-action review of the meeting and disseminated lessons from the process of organizing the meeting through the WHO’s internal knowledge-sharing networks. A set of follow-up actions and network-building activities was outlined so that the WHO team could engage and foster communities that contribute to the infodemic response.

## Results

### Meeting Participants and Discussions

A total of 1483 individuals from 111 countries and territories registered for the consultation, with 1375 and 1169 participants joining the consultation on day 1 and day 2, respectively. Registered individuals self-reported as representing the following sectors: academia or research (32%), nongovernmental organizations (21%), the private sector (17%), United Nations or intergovernmental organizations (12%), public health authorities or government (9%), health care professionals (5%), civil society (4%), and students (2%).

The presenters embodied a diverse, multidisciplinary array of thinking, providing the perspectives of the WHO, the media, government and policy makers, institutes of public health, scientific publishers, social media companies, academia, the private sector, civil society, and the humanitarian sector. They discussed approaches to studying the infodemic using conceptual, web-based, and sociobehavioral approaches, as well as social media analytics, and they demonstrated various tools and techniques designed to check facts and measure and respond to the infodemic. The discussions were grounded in lessons from the speakers’ past experiences and research.

Both the presentations and the follow-up emphasized that we are living in an interconnected world, in which the infodemic recognizes no boundaries. Information is transmitted and shared worldwide, traveling fast through social media apps and platforms, online forums, news sites, television, radio, and many other channels. Citizens exchange information more quickly than ever before, collectively experiencing in their own everyday lives the effects and changes brought about by the pandemic and the actions undertaken to respond to it. At the same time, those supporting the response are also experiencing the pandemic and infodemic through their families and communities, as well as in their professional work and networks.

### Analysis of Suggested Actions

**Figure 1:**
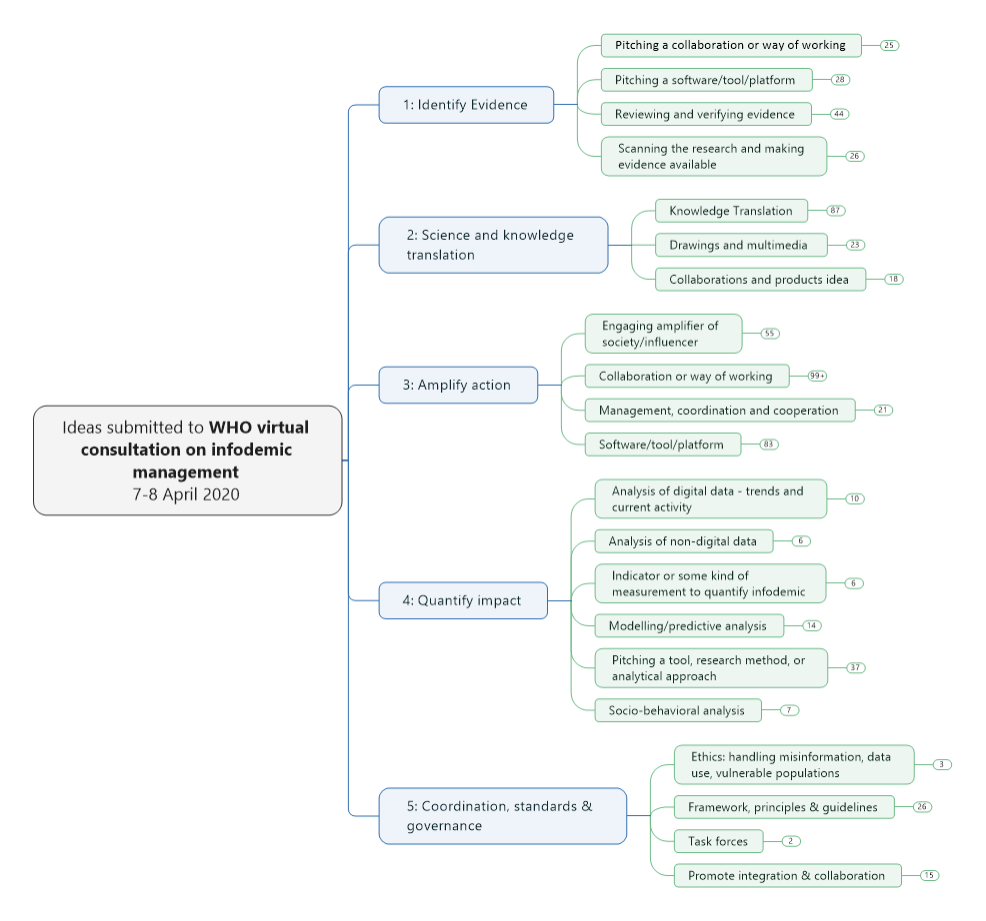
Summary categorization and count of ideas submitted by the end of the consultation period. WHO: World Health Organization.

Using a narrative analysis, we further grouped the submitted ideas into a set of five COVID-19 infodemic management areas: (1) scanning and verifying evidence (18%); (2) explaining the science (20%); (3) amplifying the reach of messages (44%); (4) measuring the infodemic and assessing trends and impacts (12%); and (5) coordination and governance (6%). The 594 collected suggestions were further summarized by theme (Figure 1).

Almost half of the proposed actions dealt with how to amplify the reach of credible messages. They suggested specific actions that the WHO or Member States could undertake, such as providing coordination and development of guidelines and frameworks; building coalitions with UN agencies, fact-checking organizations, data scientists, the AI community, social media companies, and journalists; and generating specific communication strategies and tools to reach all communities and vulnerable groups. In addition, many called for specific practical actions such as tailoring messages to specific audiences, mindful of context and literacy; developing dashboards to monitor the infodemic; developing and applying research methods to understand the infodemic at the level of information flows, populations, individuals, and communities; and analyzing the adherence to, and impact of, public health measures.

The recommendations and ideas submitted indicate that we lean on existing infodemic experience, knowledge, and tools from a range of sources, including lessons from previous outbreaks such as Zika, severe acute respiratory syndrome, and Ebola; studies on misinformation around vaccine safety and methods for addressing vaccine communication, trust, and misinformation; existing tools for reaching communities with lower internet use or literacy; and experience in community outreach and humanitarian action. At the same time, new tools were suggested to assist the process of reviewing information and taking action, and to simplify the generation and dissemination of information, messages, and materials. It was suggested that we use multidisciplinary approaches in research and analysis, such as AI for natural language processing or treatment of unstructured and text data, as well as sociobehavioral, ethnographic, and medical anthropology research methods. The submissions also called for cooperation and coordination between governments, public health authorities, journalists, fact checkers, communities, social media, the private sector, academia, and others. A detailed mind map of suggestions for action is available in Multimedia Appendix 1.

### Policy Implications

The analysis team further distilled the results of the consultation into a set of 50 proposed actions for a framework for managing infodemics in health emergencies (see Multimedia Appendix 2). The consultation revealed six implications for governments and policy makers to consider.

First, interventions and messages must be based on science and evidence, and must reach citizens and enable them to make informed decisions on how to protect themselves and their communities in a health emergency [6,7]. These basic foundations of emergency preparedness and response have been challenged and adapted in the current pandemic context by the rapid exchange of information and consequent shortened time frames for decision making.

To be able to provide policy solutions to the challenges caused by the infodemic, we must acknowledge the challenges that exist in managing the creation and dissemination of trusted information so that it is not excessive, overwhelming, or confusing and deciding when and how to counter misinformation.

- Work is required to slow down and streamline the flow of information of all kinds. This should be guided by a unified strategy for producing and disseminating trusted information, and a constantly updated set of guidelines to counter and correct misinformation using a measured approach firmly grounded in state-of-the-art sociobehavioral research. COVID-19 has resulted in an explosion of evidence generation and synthesis activities—“not only an ‘infodemic on top of the pandemic,’ but also an ‘infodemic on top of the infodemic’” [8]. These activities should be internationally coordinated to avoid duplication while guaranteeing epistemic diversity.
- As the body of evidence grows during an emergency, guidelines and messages may change or be amended. Care needs to be taken to ensure propagation of up-to-date information to communities and individuals through all appropriate channels, including to those communities for which web-based sources are not the primary source of information and those for which information needs to be adapted to local languages, literacy levels, and contexts.
- In parallel, scientific findings must be collated, reviewed, appraised, and assessed for relevance to help form recommendations and policies that have the greatest possible positive impact on the health of individuals and populations [9]. Scientific and public health institutions have central roles to play in this process. Editors of medical journals could also help manage the infodemic problem by, for example, producing plain language summaries for journalists and the public to accompany each article related to COVID-19.
- The capacity must be in place to promote trusted content; check facts; and monitor, verify, report, and respond to misinformation. Work is required to verify and counter the spread of misinformation and introduce effective regulatory approaches to limit its impact. This could be strengthened through partnerships between public health authorities, communities, journalists, fact-checking initiatives, social media platforms, and other communication channels.

Second, knowledge should be translated into actionable behavior-change messages, presented in ways that are understood by and accessible to all individuals in all parts of all societies. Cultural and contextual sensitivity in the platforms and messages used, and translation into local languages are essential. Coordinated work and partnering with a variety of stakeholders, including civil society, is required to ensure the availability of information targeted at vulnerable or hard-to-reach communities via nondigital routes. An on-the-ground network of global field workers could help reach out to highly vulnerable people to ensure they can access reliable information, as many citizens around the world still do not have access to pandemic information on the internet.

Third, governments should reach out to key communities to ensure their concerns and information needs are understood, tailoring advice and messages to address the audiences they represent. Through this process, communities of all kinds, whether neighborhood, religious, professional, or otherwise, should amplify the right public health messages in ways that are user-friendly and can lead to the right changes in behavior. For example, active engagement calls and dialogue could be established between public health authorities and private sector employers, telecom companies, the food and agriculture sector, faith-based organizations, health care and medical professional associations, and the media.

Fourth, to strengthen the analysis and amplification of information impact, strategic partnerships should be formed across all sectors, including but not limited to the social media and technology sectors, academia, and civil society. Through strategic partnerships with health authorities, these platforms can place and prioritize relevant information and advice, ensuring it is seen by citizens, helping to fact-check, measuring and describing the infodemic, tracking trends, and observing and analyzing the impact of messages and interventions on population behavior. There is a wealth of information on these platforms that can help us to understand the sentiments of different populations and guide effective public health measures. Rather than a complement to public health, the infodemic dimension should be considered a pillar of an integrative approach to public health in complex knowledge societies.

Fifth, health authorities should ensure that these actions are informed by reliable information that helps them understand the circulating narratives and changes in the flow of information, questions, and misinformation in communities. Analysis of online conversations; narratives; and TV, radio, and news media could be systematically applied and paired with fact-checking resources. Analysis of circulating narratives, knowledge, risk perceptions, behaviors, and trust at population and community levels could provide rapid snapshots to inform appropriate policy interventions. Mixed-methods research can monitor trends in acceptance of public health measures. Examples of such research methods include sociobehavioral research and sentiment and media analysis of digital information from online conversations; TV, radio, and news media; and community dipstick surveys.

Sixth, following experiences to date in responding to the COVID-19 infodemic and the lessons from other disease outbreaks, infodemic management approaches should be further developed to support preparedness and response, and to inform risk mitigation, and enhanced through data science and sociobehavioral and other research.

## Discussion

This WHO technical consultation was entirely held online, with no panelists or participants travelling to the meeting. In terms of the number of participants, it was one of the biggest meetings ever convened by the WHO—comparable to the annual World Health Assembly. It was also the first consultation to address the phenomenon of infodemic management in health emergencies. This online consultation was an effective and cost-efficient way of reaching and interacting with a large and diverse community and producing solid outcomes.

The infodemic impacts citizens in every country, and addressing it is a new and centrally important challenge in responding to the COVID-19 pandemic—and will be so for future pandemics. The consultation discussions and online brainstorming generated clear themes that can inform discussions and actions for effective management of infodemics in all countries. The participants, presenters, and experts collectively agreed that, today more than ever, it is crucially important for authorities, other stakeholders, and the public to have access to the right data and information, at the right time, and in the right formats. The better the data and information available at all levels, the smarter and more effective the response to the pandemic will be [10].

The extraordinary interdependence of the sociobehavioral dimension of the pandemic with individual and public health makes the infodemic a serious threat but is also an opportunity to shape views and behaviors in our societies. The spread of misinformation [11,12] can trigger behavioral responses that, in turn, can further expose individuals and communities to health risks. Although the impact of misinformation on society is still under scrutiny, there is increasingly convincing evidence that deliberate misinformation operations and social manipulation [13] have taken place during major events in the past—including in several high profile political instances [14]—and that they might play an important role during other high profile, strategically important or exceptional events, including the COVID-19 pandemic.

In the study of epidemics, epidemiology experienced a turning point during the 20th century through the application of mathematical and statistical methods to this scientific field. In the study of infodemics, the 21st century will see the development of infodemiology, a novel scientific discipline required to unravel the complex propagation patterns of the infodemic. In practice, infodemiology requires a transdisciplinary approach integrating applied mathematics, social, and behavioral sciences; communication and information science; digital health research; data science; and complexity science. This scientific discipline, as it addresses research priorities driven by health policy-making needs, can generate evidence to inform the development of tools, methods, and infodemic management interventions, and contribute to the monitoring of public health interventions during outbreaks, thus strengthening outbreak preparedness and response in health emergencies.

Responses to the COVID-19 pandemic and the related infodemic require swift, regular, systematic, and coordinated action from multiple sectors of society and government. It remains crucial that we promote trusted information and fight misinformation, thereby contributing to saving lives as the pandemic continues to unfold and run its course. This requires timely translation of evidence into knowledge that people can use, adapted to their local cultures, languages, and contexts. This needs to be supported by facts and analytics, backed up with constant monitoring of the impact of trusted information, and work to counteract misinformation.

We call on citizens from all parts of society to demand reliable, evidence-based information, and to take actions that empower their communities to use trusted information to protect the most vulnerable and themselves.

## Appendix

Multimedia Appendix 1 An interactive mind map of suggested actions submitted during the consultation.

Multimedia Appendix 2 A set of 50 actions for an infodemic management framework that have been consolidated from the suggestions received during consultation.

## Abbreviations

AI: artificial intelligence
CET: Central European Time
COVID-19: coronavirus disease
EPI-WIN: WHO Information Network for Epidemics
WHO: World Health Organization
