# Supplementary material for: Framework for Managing the COVID-19 Infodemic: Methods and Results of an Online, Crowdsourced WHO Technical Consultation
Source: J Med Internet Res. 2020 Jun 26;22(6):e19659. doi: 10.2196/19659 (PMC7332158; doi:10.2196/19659)
Supplement: Multimedia Appendix 1 [file jmir_v22i6e19659_app1.zip › supplement 1 interactive mindmap summary consulation ideas vertical format doi.html]

Loading...


Source Sans Pro 400

Source Sans Pro 700

Comfortaa 300

Comfortaa 700

Loading...
