# Supplementary material for: Framework for Managing the COVID-19 Infodemic: Methods and Results of an Online, Crowdsourced WHO Technical Consultation
Source: J Med Internet Res. 2020 Jun 26;22(6):e19659. doi: 10.2196/19659 (PMC7332158; doi:10.2196/19659)
Supplement: Multimedia Appendix 2 [file jmir_v22i6e19659_app2.pdf]

This is a Multimedia Appendix to a full manuscript published in the J Med Internet Res. For full copyright and citation information see http://dx.doi.org/10.2196/19659

Annex 1: Framework for managing infodemics in health emergencies

Interim draft for use during the COVID-19 response

This infodemic framework is proposed in the context of the COVID-19 response, and will be reviewed, and adjusted if necessary, after the pandemic. It is hoped that each WHO Member State and relevant actor of society will, within their mandate, apply localized infodemic management approaches adapted to national and other contexts and ongoing practices.

Action area 1: strengthening the scanning, review and verification of evidence and information

Evidence generation and synthesis

1. Develop and support international efforts to coordinate production and dissemination of evidence syntheses, and reduce duplication of effort
2. Develop tools for rapid appraisal of evidence and synthesis of knowledge, and to disseminate the information they produce
3. Develop critical appraisal tools and criteria for reporting and assessing "grey"/non-academic evidence and community grassroots responses to overcoming COVID-19.

Evidence synthesis and knowledge translation

4. Establish national rapid evidence synthesis teams containing knowledge translation specialists tasked with integrating knowledge translation into emergency health responses
5. Consider establishing mechanisms within WHO to build sustainable capacity for rapid evidence synthesis and knowledge translation, through mechanisms such as the WHO EPI-WIN network and platforms such as WHO Academy and Open WHO
6. Promote and support systematic reviews of evidence about public health and infodemic management interventions in health emergencies, to identify gaps and opportunities in research. Develop this draft framework further with a set of recommended infodemic management interventions
7. Strengthen and support community platforms that make available rapid knowledge synthesis and evidence maps, reference for localization of guidelines in Member States and analysis of uptake of WHO guidelines, and guidelines for communicating and disseminating evidence from systematic reviews.

Publication and dissemination of scientific evidence

8. Collaborate with scientific journals to define a set of principles for managing, reporting and critically appraising new evidence in order to promote public clarity of scientific

findings (such as plain language summaries for journal articles and/or virtual journal clubs)

9. Support and reinforce the Open Science values and practices of open data, open peer review, open source and open access, as well as standards for reporting evidence that enable rapid synthesis and evaluation of the evidence in systematic reviews
10. Clearly communicate the stages of the scientific peer review process, and the advantages and limitations of using pre-published articles that are rapidly shared
11. Develop tools for ranking the provenance, timeliness and credibility of scientific sources to aid citizens, media, health authorities and other scientists, so that the overview of these sources provides a kind of "evidence barometer."

Action area 2: strengthening the interpretation and explanation of what is known, fact-checking statements, and addressing misinformation

Risk communication and infodemic management

12. Consider establishing or strengthening national mechanisms in Member States for risk communication that involve multidisciplinary teams of experts from national institutes of public health, journalists, the fact checking and misinformation-fighting community, monitoring and analytical experts and other relevant actors in a coordinated effort to disseminate verified information and respond to misinformation
13. Tailor messages to targeted audiences based on available evidence, and debunk the most harmful myths (e.g. through the WHO EPI-WIN network); and develop approaches, standards and tools that address the changing of messages and guidance as knowledge about the pathogen and the disease increases
14. Coordinate efforts to produce reliable, multilingual content in response to claims and questions about preventive measures and treatments, and base the work on research about what questions are circulating in communities.
15. Foster dialogue and communication between public health organizations and local journalists to strengthen visibility and trust across professional sectors and raise the capacity of local media to use verified information
16. Consider strengthening journalists' training on health and scientific topics; using Q&As with respected media trainers and health experts for training of journalists; and incorporating retractions of unconfirmed or unfounded statements into standard reporting practice.
17. Define and promote a research agenda on risk communication in the digital age to develop scalable interventions that can address the receptivity of individuals and the sharing of misinformation online.

Development of trusted sources, factchecking, and response to misinformation

18. Develop tools and guidance to promote risk communication, disseminate trusted information and respond to misinformation during the COVID-19 pandemic and other health emergencies. These could include (but should not be limited to):
    - Guidelines and tools on use of digital tools and analytics for risk communication and community engagement in health emergencies

    - checklists and guidance on how to promote trusted content and respond to misinformation
    - Protocols to decide which stories need to be debunked because they are gaining traction and approaching a strategic tipping point
    - Resources for citizens to promote digital health and media literacy
19. Support the development of networks of trusted sources of information and networks for standards-based, multilingual factchecking activities and misinformation response
20. Develop tools and standards for assessing the integrity/accountability of factchecking initiatives, including a common glossary and terminology for describing the infodemic and its elements to facilitate communication, exchange of information and management of the infodemic across all levels of society
21. Build capacity for promoting trusted content and fact-checking, monitoring, verifying, reporting and responding to misinformation, by developing a network of WHO Collaborating Centres and providing courses on training platforms such as Open WHO
22. Support collaborative development of integrated resources on communication in public health emergencies, including but not limited to:
    - A global resource centre and dashboards for fact-checking and misinformation that provide an integrated overview of information and related activities
    - Infodemic dashboards for emergencies, but also for more slow-burning systematic issues such as vaccine mistrust and misinformation, incorporating behavioural and other multidisciplinary analyses of past experiences
23. Support the propagation of updated information through innovation in information networks and the facilitation of collaborative, distributed factchecking activities.

Social media, web and other communication channels

24. Engage social media companies and other locally dominant channels of information dissemination in promoting access to trusted health information and reducing the impact of misinformation
25. Ensure that social media platforms act to support and innovate the dissemination of trusted health information and respond to the propagation of misinformation on their platforms. Actions to this effect could include:
    - Improving the alignment of platforms' terms of use to local information laws in order to address disinformation/misinformation
    - Implementing mechanisms for user-reported misinformation alerts, to facilitate faster review of misinformation
26. Work with domain registration companies to review any new domain registrations related to COVID-19
27. Ensure that organizations with established and functioning websites do not register new domains for the pandemic, because this makes it difficult to gain traction in search algorithms. Instead, organizations should dedicate a page or section on their already existing websites to COVID-19
28. Innovate to provide web readers with a "likelihood of fakeness" assessment of information based on machine learning and integrated repositories of misinformation and trusted content.

Action area 3: strengthening the amplification of messages and actions from trusted actors to individuals and communities that need the information

Coordination of information dissemination

29. Coordinate the dissemination of information to reduce the proliferation of sources
30. Build intersecting platforms to share concrete communications practices and resources by sector (e.g. for governments, journalists, health care professionals, the technology sector, community leaders, law enforcement, students, and others), fostering self-learning and the exchange of information.

Localization of messages and community engagement

31. Foster networks and communities for localization, context adaptation, and translation of communication material, and link up with content production and dissemination networks
    - Involve, and share leadership with, knowledge producers, journalists, librarians, policymakers, civil society and local leaders
    - Where these networks do not exist, engage, and share leadership with, local health care centres, community health workers and/or civil society, with the aim of cascading information down to individual level
32. Devise and implement approaches that incentivize society to engage with WHO-recommended content. Methods for doing this might include memes, games, cartoons, quizzes, surveys, polls, competitions, participation in podcasts, scientific entertainment programmes and other events. These should be executed using a coordinated approach across social media, mobile, web, email, radio, TV, and other channels down to word of mouth, and should include the use of influencers or other trusted mediums.

Use of communication channels

33. Ensure the strategic use of all relevant communication channels to disseminate information, including social media, news, radio and/or community and other leaders. Include community mechanisms for health provision, psychosocial support, education, provision of water, sanitation and hygiene (WASH), and vaccine safety communication/promotion of immunization demand
34. Produce tools and guidance on how to engage social media platforms, and use hashtags and other practices to disseminate health information as effectively as possible
35. Collaborate with private sector communications platforms (social media, communication boards/online forums, messaging apps, etc.) to disseminate health information and engage audiences through methods including Q&As, interactive sessions and the use of bots for content dissemination
36. Ensure that social media platforms develop policies that institutionalize their support for efforts to share information from WHO, UN agencies, national authorities and other trusted sources

37. In low-resource settings with low internet penetration, consider using text messaging and Interactive Voice Response (IVR) to disseminate messages and collect feedback from the population.

Health, digital health and media literacy

38. Implement programmes to boost critical thinking skills and health, media and digital health literacy among the population, building capacity to discern what information is reliable
39. Work in partnership with the education sector, health literacy experts and others to develop curricula, guidance, tools and evidence to promote digital health and health and media literacy across the population throughout the life course, as well as specifically among health care workers and vulnerable populations.

Action area 4: strengthening the analysis of infodemic*s*, including *analysis of* information flows, monitoring *the* acceptance of public health interventions, and *analysis of* factors affecting infodemic*s* and behavio*urs* at individual and population levels

Develop monitoring of the infodemic

40. Monitor, analyze and evaluate the implementation of infodemic management interventions
41. Promote and develop new data sources, methods and approaches for analysis of infodemic management interventions
42. Develop a running research agenda for monitoring, analysis and evaluation of infodemic components and interactions, and infodemic management interventions
43. Develop and introduce monitoring of key indicators for questions, opinions and attitudes to inform infodemic interventions, including information from vulnerable and at-risk groups; develop new indicators for monitoring infodemic management from the points of view of policymakers, the general public, health care workers, individuals, and particular communities
44. Develop a multidisciplinary research agenda and develop methods, data sources and mixed-methods analysis protocols for measuring different aspects of the infodemic, including in the areas of:
    - Information flows in digital and traditional media, including analysis of narratives, questions being asked, sentiment, web search activity and information dissemination networks
    - Analysis of information flows focusing on the reliability of information vs types of misinformation, and exchanges of trusted information and misinformation
    - Trust and credibility measurement, including people's attitudes towards information, its sources, and what system it comes from; how these lead to trust or mistrust; and prediction of the likelihood of action
    - Audiences' interactions with information, including their vulnerability to misinformation, misinformation exposure, and self efficacy (a person's belief in having ability to change own behavior, beliefs, motivation, to counter misinformation, and take up knowledge that leads to healthy behaviour and

recognize misinformation/low quality information) as related to their health literacy, beliefs, knowledge, and behaviour

- Analysis to inform the implementation and revision of infodemic management interventions
- Analysis of circulating information and beliefs, and trust dynamics at community level
- Modelling of infodemic risk at societal level.

Develop research on health information dissemination and uptake

45. Develop infodemic research priorities to identify enablers of, and barriers to, the availability of trustworthy health information, including how to improve production and dissemination of evidence-based information for the public, patients, and health professionals, and measures to increase health literacy and the ability to find and interpret such information.

Action area 5: strengthening systems for infodemic management in health emergencies

46. Consider establishing national coordination mechanisms or task forces in Member States to coordinate all aspects of infodemic management in support of risk communications, misinformation response, community engagement and measurement of the impact of infodemic interventions.
47. Following the experience of the response to the COVID-19 infodemic, and the lessons of other disease outbreaks, refine this draft infodemic management framework to support preparedness and response in the future and inform risk mitigation, enhancing it through data science, socio-behavioural and other research. This could include, but need not be limited to:
    - Building capacity, shared open tools, and collaborations across sectors in global, national, and community responses
    - Production of a value statement declaring access to correct health information as a basic human right and promoting dissemination of accurate health information (including up-to-date information that has been localized for specific communities) and fact checking and monitoring of misinformation
    - Developing guidelines on ethical considerations for analysis and design of infodemic interventions
48. Build a network of WHO Collaborating Centres for all aspects of infodemic management, and pursue collaborations with other UN agencies that are doing infodemic monitoring and management work in the field, to conceive and deliver capacity building programmes online and in Member States
49. Promote open source tools and standards that ensure reusable analysis and interoperable exchange of infodemic data, AI training datasets and models
50. Use innovative methods, such as hackathons, innovation challenges and online brainstorms, to collect further ideas and innovations, and crowdsource problem-solving in infodemic management.
